# Performance of community health workers managing malaria, pneumonia and diarrhoea under the community case management programme in central Uganda: a cross sectional study

**DOI:** 10.1186/1475-2875-13-367

**Published:** 2014-09-18

**Authors:** James Bagonza, Simon PS Kibira, Elizeus Rutebemberwa

**Affiliations:** Department of Health Policy, Planning and Management, School of Public Health, Makerere University College of Health Sciences, Kampala, Uganda; Department of Community Health and Behavioral Sciences, School of Public Health, Makerere University College of Health Sciences, Kampala, Uganda

**Keywords:** CHWs, iCCM programme, Performance, Evaluation, Uganda

## Abstract

**Background:**

Lay community health workers (CHWs) have been widely used to provide curative interventions in communities that have traditionally lacked access to health care. Optimal performance of CHWs managing children with malaria, pneumonia and diarrhoea in communities is desired if a reduction in childhood morbidity and mortality is to be achieved. This study assessed factors influencing performance of CHWs managing malaria, pneumonia and diarrhoea under the Integrated Community Case Management (iCCM) programme in Wakiso district, central Uganda.

**Methods:**

A cross sectional study was conducted among 336 CHWs. Data was collected using interviews and record reviews. Performance was measured using composite scores based on the core activities of CHWs under the iCCM programme. These core activities included: treating children under five years, referring severely sick children including newborns, home visits, counseling caregivers on home care, record keeping and community sensitization. Descriptive and inferential statistics using odds ratios were done to determine factors influencing performance of CHWs.

**Results:**

Of the 336 respondents, 242 (72%) were females and the overall level of good performance was 21.7% (95% CI, 17.3-26.1%). Factors significantly associated with performance were: sex (females) (AOR 2.65; 95% CI, 1.29 -5.43), community support (AOR 2.29; 95% CI, 1.27-4.14), receiving feedback from health facilities (AOR 4.90; 95% CI, 2.52-9.51) and having drugs in the previous three months (AOR 2.99; 95% CI, 1.64-5.42).

**Conclusion:**

Only one in every five CHWs performed optimally under the iCCM programme. Strategies to improve drug supply, community support and feedback provision from the formal health system are necessary to improve the performance of CHWs.

## Background

Over the past couple of decades, studies have shown that community health workers (CHWs) can help reduce morbidity and mortality in settings that have traditionally lacked access to health care [1, 2]. The intermediation of CHWs in healthcare delivery is widening as they are crucial in increasing universal access to healthcare provision and the attainment of the Millennium Development Goals [3].

Community health workers are men and women chosen by the community, and trained to deal with individual and community health problems, working in close relationship with the formal health care system [4]. They should have basic literacy and numeracy levels [4]. CHWs are considered as a third health service delivery work-force and have evolved with community-based healthcare programmes [5]. However, their titles, profiles and deployment vary across countries, conditioned by their aspirations and economic capacities [6, 7].

In an attempt to reduce child morbidity and mortality, Uganda adopted the integrated community case management (iCCM) policy and lay CHWs are main drivers of this strategy. Under this policy, CHWs are trained and supported to assess, manage and refer when appropriate, children under five years with malaria, pneumonia and diarrhoea [8, 9]. Each CHW takes care of 25 to 30 households and is supplied with amoxicillin for treating non severe pneumonia, artemisinin-based combination therapy (ACT) for uncomplicated malaria; low osmolarity oral rehydration salts (ORS) and zinc for diarrhoea. These three diseases are the leading causes of child ill health, and death in Uganda [10]. The approach further complements health facility-based management of common childhood illnesses by offering timely management of illnesses within 24 hours of onset of symptoms at the community level [11].

Haines and colleagues [1] propose three determinants of the success of a CHW programme. These include: community factors such as location, support, respect, health beliefs, national socioeconomic and political factors, including corruption and political will and health system factors such as remuneration, training and supervision [1, 12]. CHWs require supportive supervision, clearly defined roles with specific tasks, locally relevant incentive systems that combine monetary and non-monetary incentives, recognition, training opportunities, community and policy support, and strong leadership [13]. All of these factors can play a role in the length of time a CHW serves thus affecting their performance. Social demographic characteristics, such as age, sex, marital status and education level, greatly influence performance of CHWs. In Bangladesh, CHWs who were married and with good educational background perform better since they are respected by the community [14]. In Nigeria, CHWs with higher education were easier to train and had a better grasp of skills needed to diagnose and treat common illnesses [15]. While the benefits of having basic education are obvious, evidence from Peru, showed that CHWs with higher education had opportunities for alternative employment and tended to migrate from one job to another [16]. However a study in western Uganda showed that age, sex and educational level were not associated with the ability of CHWs to diagnose pneumonia [17].

Additionally, individual CHW motivation impacts on retention and performance. Motivation is driven by many elements including intrinsic factors such as an individual's work-related goals, as well as his/her sense of altruism, self-efficacy, and organizational commitment. Extrinsic factors include peer approval, incentives provided, and expectation of future paid employment [18–20]. These are similar to the factors found to affect motivation and performance of formally trained health workers in low income countries in recent reviews [21, 22].

Few studies have investigated the performance of CHWs managing malaria, pneumonia and diarrhoea in children under the iCCM programme [12, 23, 24]. These studies have mainly focused on CHWs who only treat malaria and pneumonia excluding diarrhoeal illnesses and are inconclusive on factors that affect their performance. The aim of this study was to determine the performance of CHWs managing common childhood illnesses and to assess factors influencing their performance so to guide future scale up of the iCCM programme.

## Methods

### Study setting

This study was conducted in Busiro east and Kyandondo north Health Sub districts (HSDs) in Wakiso district. Wakiso is located in central Uganda, about 12 km from Kampala, the capital city. It has a population of 1,260,900 [10] and the top five major causes of morbidity in the district are malaria, respiratory tract infections, intestinal worms, skin diseases and diarrhoea [10]. The district has about 1,400 CHWs trained and supported to manage children with malaria, pneumonia and diarrhoea in communities. Each CHW takes care of about 25 to 30 households. There are seven HSDs which include Busiro east, Busiro north, Busiro south, Kyadondo north, Kyadondo east, Kyadondo north and Entebbe. A health sub-district is the functional zone of the district health system responsible for delivery of the Minimum Health Care Package (MHCP). The MHCP services include preventive services such as childhood immunization, health promotion and education as well as treatment and control of common infectious diseases such as malaria, HIV/AIDS and Tuberculosis. The HSD has a catchment area comprising several peripheral health facilities whose health workers supervise CHW’s activities.

### Study design

This was a cross sectional study that employed quantitative methods of data collection and the outcome of interest were measures of performance of CHWs under the iCCM programme. Eligible respondents were CHWs who had been recruited on the iCCM programme for more than six months by the time of the study and gave informed consent. CHWs who had travelled out the district or not available by the time of the study were excluded.

### Sample size and sampling procedure

A sample size formula for cross-sectional studies was used to calculate the required number of CHWs to participate in the study [25]. The study sample of 340 CHWs was based on the following assumptions: a two sided test with a precision of 5%, and level of performance of CHWs from a previous study was 33% [26]. On average each health sub district had 180 CHWs managing children under the iCCM programme and thus all CHWs in the selected two HSDs were included. Busiro east and Kyadondo north were selected randomly out of the seven HSDs in Wakiso district. In each HSD, a list of all sub counties and town councils was obtained from the district focal person responsible for CHW activities. In each sub-county or town council, Lists of CHWs managing childhood illnesses were obtained from health facilities and located CHWs through their supervisors and local council leaders.

### Study tools and data collection

Semi-structured interviewer administered questionnaires and data extraction sheets were used to collect quantitative data. Data collection tools were translated into local language (Luganda) and back translated for consistency of meaning. The study instruments sought to collect informamation on respondent’s socio-demographic characteristics, performance of iCCM activities, community and health system factors. The tools were pretested in two villages outside the study area and modified accordingly. Interviews and record reviews were used to collect data on core iCCM programme activities and performance indicators. The core iCCM programme activities included treating children less than five years presenting with cough or fever or diarrhoea, referring severely sick children, counseling mothers on home care, home visits (for newborns) and referring a sick neonate to health facilities. Others included community sensitization and following up children discharged from health facilities.

For the iCCM programme indicators, we collected data on number of children seen per month and quality of CHW reports in terms of timeliness and completeness. CHWs records were reviewed for the previous six months by the time of the study. This information was got from the iCCM sick patient register for children below five years. Completeness of a monthly report was based on the following general information: date, patient name, sex, age and respiratory rate. Other details of the reports were not considered since they depended on the patient diagnosis and this was not always uniform for all patients. This was done to minimize the measurement bias among the data collectors. Data on timeliness was based on the CHW’s monthly reports that were received at the supervising health facility. Timeliness was defined as the submission of iCCM reports to a supervising health facility before the 7^th^ day of the following month [27, 28].

Semi-structured questionnaires also captured data on individual CHW characteristics, community and health system factors that might affect CHW performance. Community factors included; recognition, respect, community support, immediate family support and support from local leaders. To assess support from community, local council leadership and immediate family support, CHWs were asked to rate their perceptions of the support they received according to this scale: very high/good, high/good, fair, low/poor and very low/poor. During data management, responses which indicated very high/good or high/good support were categorized as high/good support while responses that indicated fair, low/poor or very low/poor support were combined and categorized as low/poor support. Health system factors included: CHW trainings, receiving appointment letters, supervision, workload, resources/supplies, policy/guidelines, job aids, feedback provision, reporting, facilitation, in-kind incentives and drug availability.

### Measurement of performance

Performance was assessed using composite scores [29]. For the core iCCM activities, scoring was done based on when each activity was last performed by each CHW on the programme [29, 30]. Activities done within the past one week were scored four points while those done in the past two weeks, three weeks and four weeks were scored three points, two points and one point respectively. Activities that were done more than four weeks ago attracted no points [29, 30].

The composite score ranging from 0 to 28 points was the sum of the scores for all the seven activities. For the iCCM programme indicators, two key indicators (quality of iCCM data and number of children seen per month per CHW) were assessed. For the quality of iCCM data, two attributes were assessed: timeliness and completeness. Reports for the last six months were reviewed and timeliness was defined as the submission of iCCM reports to the supervising health facility before the 7^th^ date of the following month (reporting period). A report was regarded as complete if all the components/fields were filled. A score of one was given to each timely and complete report, a similar approached was used to study the performance of CHWs in a primary health care programme in Zambia [26]. For the number of children seen, CHWs who saw more than 10, between 7-10, 3-6 and less than three children in a month scored 4, 3, 2, 1 and zero points respectively. This was similar to previous evaluations of Home based Management of Fevers (HBMF) programme done in northern Uganda [31]. The scores ranged from 0 to 36 from these two attributes. Individual sum of the composite scores for each CHW were expressed as a percentage of the total maximum score expected. Performance of 0 to 74.9% was classified as poor while CHWs who scored 75% and above were classified as good performers [29].

### Data management and analysis

Data were double entered in Epi-Info software version 3.3.2 and analysed using STATA 10 (STATA Corp, College Station, TX, USA). CHWs characteristics and performance were summarized using descriptive characteristics. Performance was dichotomized as good for scores of 75% and above and poor for scores less than 75%. To identify factors associated with performance of CHWs, A multi-variable logistic regression analysis was done, with performance as the binary outcome. Before conducting multivariable analysis, the existence of multicolinearlity was investigated using the correlation coefficient between each pair of the independent variables. Correlated variables (correlation coefficient value greater than 0.5 with a p-value less or equal to 0.05) were excluded from the logistic regression analysis model. All variables with a p-value of less than 0.2 [29] at univariate analysis as well as variables known to predict performance of CHWs, such as sex, education level and marital status, from literature [14, 15] were used in multivariate analysis. A binary logistic regression analysis with a stepwise elimination approach was done to determine independent factors associated with performance of CHWs. Conclusions were drawn based on the adjusted odds ratios with their corresponding 95% confidence intervals. Model robustness was checked by Wald chi square.

### Ethical considerations

Ethical approval was obtained from Makerere University School of Public Higher Degrees Research and Ethics Committee and Wakiso District Health authorities. Written informed consent was obtained from the study participants. Confidentiality was censured by securely keeping data at all times and no personal identifiers were used or recorded on study tools.

## Results

### Characteristics of respondents

Table 1 shows background characteristics of CHWs. Four of 340 study participants were excluded from analysis due to incomplete data. Of the 336 respondents, 72% were females and 77.6% were above 35 years of age while 43.2% had attained secondary level education.

**Table 1:** **Background characteristics of CHWs**

Characteristic		Respondents
		[n, (%)]
**Sex**	Males	94 (28.0)
	Females	242 (72.0)
**Age**	Less or equal to 35 years	76 (22.6)
	More than 35 years	260 (77.4)
**Education**	Primary	154 (45.8)
	Secondary	145 (43.2)
	Tertiary	37 (11.0)
**Religion**	Catholics	116 (34.5)
	Anglicans	159 (47.3)
	Moslems	44 (13.1)
	Others	17 (5.1)
**Marital status**	Never married	24 (7.1)
	Married	241 (71.7)
	Separated/divorced	42 (12.5)
	Widowed	29 (8.6)
**Occupation**	Unemployed	60 (17.9)
	Formal employment	41 (12.2)
	Self employed	235 (69.9)
**HSD**	Busiro East	169 (50.3)
	Kyadondo North	167 (49.7)

### Performance of community health workers

Table 2 shows the different levels of performance among respondents. Community health workers who scored 75% and above were classified as good performers while those who scored below 75% were poor performers. The overall level of good performance among respondents was 21.7% (73, 95% CI = 17.3 – 26.1%). A majority of the respondents 65.5% (220/336) scored between 50% and 74% while only 12.5% (43/336) scored between 25 and 50%. About a quarter of female respondents 25.2% (61/242) and 37.8% (14/37) of participants with tertiary education as their highest level of education attained were good performers. Nearly one fifth, 20.4% (48/235) of respondents who were self employed were good performers.

**Table 2:** **Level of performance of CHWs**

Variable	Performance categories		
	Score (25-49%)	Score (50-74%)	Score (75+)
	[n (%) N = 43]	[n (%) N = 220]	[n (%), N = 73]
**Age**			
≤35 years	13 (17.1)	46 (60.5)	17 (22.4)
>35 y ears	30 (11.5)	174 (66.9)	56 (21.5)
**Sex**			
Male	15 (16.0)	67 (71.2)	12 (12.8)
Female	28 (11.6)	153 (63.2)	61 (25.2)
**Education**			
Primary	27 (17.5)	99 (64.3)	28 (18.2)
Secondary	13 (9.0)	101 (69.7)	31 (21.4)
Tertiary	3 (8.1)	20 (54.1)	14 (37.8)
**Employment**			
Unemployed	9 (15.0)	37 (61.7)	14 (23.3)
Formal employment	3 (7.3)	27 (65.9)	11 (26.8)
Self employed	31 (13.2)	156 (66.4)	48 (20.4)
**Health sub district**			
Busiro east	17 (10.1)	118 (69.8)	34 (20.1)
Kyadondo north	26 (15.6)	102 (61.1)	39 (23.4)
**Overall performance**	**43 (12.8)**	**220 (65.5)**	**73 (21.7)**

None of the respondents scored less than 25%.

### Associations between background characteristics and performance of CHWs

Table 3 shows results from bivariate analysis of associations between individual factors and performance of CHWs. Factors found to be significantly associated with good performance were: being female (OR 2.3; 95% CI, 1.17- 4.54) and tertiary education level (OR 2.74; 95% CI, 1.23 – 6.08). Age, religion, employment and marital status were not significantly associated with performance at unadjusted analysis.

**Table 3:** **Association between demographic characteristics and performance of CHWs**

Variable	Performance		Crude analysis	
	Good (n, %)	Poor (n, %)	OR (95% CI)	P value
**Sex**				
Males	12 (16.4)	82 (31.2)		
Females	61 (83.6)	181 (68.8)	2.3 (1.17-4.54)	0.013
**Age of respondents**				
<=35 years	17 (23.3)	59 (22.4)		
>35 years	56 (76.7)	204 (77.6)	0.95 (0.51-1.76)	0.877
**Education level**				
Primary	28 (38.4)	126 (47.9)		
Secondary	31 (42.5)	114 (43.3)	1.22 (0.69-2.17)	0.488
Tertiary	14 (19.2)	23 (8.7)	2.74 (1.23-6.08)	0.010
**Religion**				
Catholic	26 (35.6)	90 (34.2)		
Anglicans	34 (46.6)	125 (47.5)	0.94 (0.53-1.68)	0.838
Moslem	7 (9.6)	37 (14.1)	0.65 (0.26-1.65)	0.365
Other	6 (8.2)	11 (4.2)	1.89 (0.63-5.65)	0.247
**Marital status**				
Never married	810.9)	16 (6.1)		
Married	52 (72.6)	189 (71.9)	0.55 (0.22-1.36)	0.190
Separated/divorced	10 (13.7)	32 (12.2)	0.63 (0.2-1.92)	0.407
Widow/widower	3 (4.1)	26 (9.9)	0.23 (0.05-1.08)	0.042
**Employment status**				
Unemployed	14 (19.2)	46 (17.5)		
Formal employment	11 (15.1)	30 (11.4)	1.2 (0.48-3.02)	0.691
Self employed	48 (65.8)	187 (71.1)	0.84 (0.43-1.66)	0.622

### Associations between community factors and performance of CHWS

Table 4 shows results of unadjusted analysis. Associations between community factors and performance of CHWs were assessed. Factors found to be significantly associated with performance on bivariate analysis were: support from: the community (OR 3.07; 95% CI, 1.76-5.38), local council leadership (OR 1.82; 95% CI, 1.05-3.13), and immediate family members (OR 2.78; 95% CI, 1.12 – 6.67) support from immediate family members and respect.

**Table 4:** **Association between community factors and performance of CHWs**

Variable	Performance		Crude analysis	
	Good (n, %)	Poor (n, %)	OR (95% CI)	P value
**Selection to become CHW**				
Local council chairperson	13 (17.8)	40 (15.2)	1.00	
Local council executive	16 (21.9)	67 (25.5)	0.73 (0.32-1.69)	0.467
Meeting of the whole village	44 (60.3)	156 (59.3)	0.87 (0.43-1.77)	0.695
**Community support**				
Poor	25 (34.2)	162 (61.6)	1.00	
Good	48 (65.8)	101 (38.)	3.07 (1.76-5.38)	<0.001
**Support from LC leadership**				
Low	43 (58.9)	190 (72.2)	1.00	
High	30 (41.1)	73 (27.8)	1.82 (1.05-3.13)	0.029
**Duration of working as CHW**				
Less than 12 months	9 (12.3)	49 (18.6)	1.00	
More than 12 months	64 (87.7)	214 (81.4)	1.63 (0.76-3.50)	0.208
**Support from immediate family**				
Low	6 (8.2)	52 (19.7)		
High	67 (91.8)	211 (80.2)	2.78 (1.12-6.67)	0.021
**Community recognition of services**				
No	3 (4.1)	17 (6.5)	1.00	
Yes	70 (95.9)	246 (93.5)	1.61 (0.46-5.56)	0.453
**Respect by community**				
Low	19 (26.1)	105 (39.9)	1.00	
High	54 (73.9)	158 (60.1)	1.89 (1.05-1.33)	0.030

### Associations between health systems factors and performance of CHWs

Table 5 shows results of unadjusted analysis showing associations between health system factors and performance of CHWs. Factors found to be significantly associated with performance were: having an appointment letter (OR 3.57; 95% CI, 2.00-6.25), workload (OR 0.45; 95% CI, 0.24-0.83) and regular submission of monthly reports (OR 5.75; 95% CI, 1.70-19.34). Others factors found to be significant were: receiving feedback (OR 4.34, 95% CI, 2.28-8.24) and having not experienced drug stock outs in the previous three months (OR 2.63; 95% CI, 1.53-4.54).

**Table 5:** **Association between health system factors and performance of CHWs**

Variable	Performance		Crude analysis	
	Good [n, %]	Poor [n, %]	OR (95% CI)	P value
**Received iCCM training**				
Yes	73 (100)	262 (99.6)		
No	0	1 (0.4)	**-**	0.598
**Refresher training in iCCM**				
Yes	64 (87.7)	236 (89.7)	1.00	
No	9(12.3)	27 (10.3)	1.23 (0.55-2.75)	0.614
**Received appointment letter**				
No	39 (53.4)	211 (80.2)	1.00	
Yes	34 (46.6)	52 (19.8)	3.57 (2.00-6.25)	<0.001
**Received job aids**				
Yes	73 (100)	255 (96.6)		
No	0	8 (3.4)	-	0.132
**Workload**				
Okay	57 (78.1)	162 (61.6)	1.00	
Too much	16 (21.9)	101 (38.4)	0.45 (0.24-0.83)	0.009
**Facilitation given**				
Inadequate	53 (72.6)	194 (73.8)	1.00	
Adequate	20 (27.4)	69 (26.2)	1.06 (0.59-1.9)	0.843
**Support from health facilities**				
Yes	68 (91.2)	222 (84.4)	1.00	
No	5 (8.8)	41 (15.6)	0.40 (0.15-1.05)	0.055
**Feedback from supervisors**				
More than a month	15 (21.5)	138 (52.9)	1.00	
Within a month	58 (79.5)	123 (47.1)	4.34 (2.28-8.24)	<0.001
**Attendance of review meetings**				
Every month	11 (15.1)	37 (14.1)	1.00	
Every 3 months	57 (78.1)	219 (83.4)	0.88 (0.42-1.83)	0.723
Once a year	5 (6.8)	7(2.5)	2.4 (0.62-9.37)	0.193
**Drug stock outs in last 3 months**				
Yes	37 (50.7)	192 (73.0)	1.00	
No	36 (49.3)	71 (27.0)	2.63 (1.53-4.54)	<0.001

### Independent factors associated with performance of CHWs

Table 6 shows results of the multivariate analysis of independent factors predicting good performances among CHWs. When factors were fitted in a logistic regression model for multivariate analysis, 99.5% (n = 334) of respondents were retained in the analysis. There was no selective elimination of respondents with regard to important demographic characteristics. Factors independently associated with good performance after controlling for confounders were: Sex (females) (AOR 2.65; 95% CI, 1.29-5.43), community support (AOR 2.29; 95% CI: 1.27-4.14), receiving feedback from health facilities (AOR 4.90; 95% CI, 2.52-9.51) and having drugs in the previous three months (AOR2.99; 95% CI, 1.64-5.42).

**Table 6:** **Logistic regression analysis for independent predictors of performance**

Variable	Crude OR (95% CI)	Adjusted OR (95% CI)	P -value
Females	2.30 (1.17-4.54)	2.65 (1.29-5.43)	0.007
Community support	3.07 (1.76-5.38)	2.29 (1.27-4.14)	0.006
Feedback from supervisors	4.34 (2.28-8.24)	4.90 (2.52-9.51)	<0.001
Drug availability in the previous 3 months	2.63 (1.53-4.54)	2.99 (1.64-5.42)	<0.001
Monetary facilitation	1.58 (0.39-6.25)	1.45 (0.32-6.44)	0.626

## Discussion

The results show that performance of CHWs was suboptimal based on the criteria used [29, 30]. Being a female, having community support, supervision and drug availability improved performance of CHWs. Only one in every five CHWs performed optimally. This level of performance is quite low given the fact that iCCM programme is still new in Uganda. Alam and others, found a similar level of performance among female CHWs in maternal and child health (MCH) activities in urban slums of Dhaka in Bangladesh [29]. However some studies suggest varied levels of good performance among CHWs ranging between 20% and 70% [6, 26]. Such variations are due to programme design, implementation, incentives given to the CHWs and community contexts. Generally, CHWs tend to perform better when the incentives they receive both monetary and non-monetary are considered reasonable, regular and sustainable. The low level of performance may be attributed to many people being involved in various economic activities as this is a semi-urban area and hence giving less attention to CHW activities.

Females CHWs performed better than their male counterparts, which is consistent with other studies that found female volunteers delivering more effectively than males [24], an impression common among policy makers as well [32]. While this may be true for MCH interventions [22], the role of male CHWs, especially in the control of epidemics in the past has been substantive across countries [14, 16]. This result could be because males are main bread winners for most households thus may not effectively work in voluntary roles given the economic demand. Further, most children under five in the districts are dependent on their mothers and thus women may find it more comfortable seeking care from fellow women which may affect male performance. Implementing iCCM may demand that the socio-cultural context of the area be put into consideration so that the persons likely to be available in that particular context be encouraged to participate.

In this study, community support was one of the most important motivators of performance of CHWs. This depends on how the community accepts, cooperates and appreciates the services offered by CHWs in communities [29, 33]. Communities tend to consult CHWs on many health issues since they believe they are trained and linked to the formal health system [14]. This not only increases their social standing and prestige but also earns them respect in community. In general, conducive and enabling environments are a pre-requisite for one to effectively and efficiently work with communities. In Bangladesh and India studies showed that community support and respect were intangible incentives to CHWs [14, 29].

Similar findings from Columbia revealed that CHWs ranked “having influence in the community” as the most important extrinsic reward that affected their performance [34]. This form of incentive may be feasible, manageable and sustainable since most governments and non-governmental organizations dealing with community health programmes often times cannot sustain regular monetary incentives. However, the extent to which communities support, such programmes depends on whether they are involved at inception and implementation of such programmes and the perceived benefits expected to accrue from these interventions [24].

Provision of feedback was an important component of the iCCM strategy in this study, yet nearly a half of the CHWs did not receive timely feedback from their supervisors. A similar study in Mali also found out that regular supervision was a key predictor of good performance of CHWs [6]. However, contradicting findings in a Zambian study showed that support supervisions did not influence the performance of CHWs [26] although in Zambia, support supervisions were irregular and there no standard method or checklist was used during supervisory visits. Under the iCCM programme, the main purpose of such feedback is to provide ongoing support, identify best practices, challenges and coping mechanisms for CHWs. Weak and inconsistent provision of feedback was cited in this study and this may be partly attributed to limited resources, mainly transport to conduct monthly supervision. Such home-based interactions with supervisors help to reinforce CHW’s competencies in case management, drug storage and record keeping as well as identifying and controlling misuse of drugs. Reliance on quarterly meetings to provide feedback is not enough as they are often too big and usually focus on discussing incentives and general challenges. Such may not necessarily lead to improvement in individual performances.

Drug availability is critical to the success of the iCCM programme. In this study, nearly two-thirds of the CHWs experienced stock outs of at least one of the medicines, mostly Amoxicillin in the previous three months. An evaluation to assess the performance of CHWs in a primary health care programme in Zambia [26] revealed similar findings where drug stock out periods ranged from six months to two years and the erratic supply of medicines was responsible for poor performance. Though the iCCM programme is a child survival strategy initiated by government, its programmatic implementation heavily relies on donor funding mainly from UNICEF and Malaria consortium [9, 31]. Such support often fluctuates and this leads to breakages in the drug supply chain system. Secondly the capacity of CHWs to adequately quantify, forecast and order for medicines to sustain them throughout the reporting periods may be lacking thus reliance on the push system with its challenges might be a realistic option for now. The fact that most children present with cough and fever coupled with the inability of some CHWs to count respiratory rate due to break down of respiratory timers, leads to over-prescription of amoxicillin, a drug most frequently out of stock. Irrational use of medicines by CHWs can result in incomplete recovery, development of resistance and adverse medicine reactions and over all failure of the programme. In general, the respect and status of CHWs in their communities unquestionably increases when they have drugs in stock and indeed there is growing evidence that suggests that the credibility of CHWs suffers when drug supplies are irregular [6, 14, 26]. Communities tend to attach more value to the curative than preventive roles of CHWs because of the immediate and tangible benefits associated. Thus absence of drugs may render the CHWs services to be perceived as irrelevant or unpopular, which affects their motivation and performance.

Although the opinions of CHWs regarding the adequacy of incentives were not statistically associated with performance, monetary facilitation was one of the highly ranked incentives that would enable CHWs perform better. Although CHWs are expected to perform on a voluntary basis, Ministry of Health guidelines on their operations provide for allowances. In fact a minimum monthly stipend of (UGX 10,000), about 4 USD paid quarterly has been suggested [8]. These guidelines further stipulate that CHW activities should be planned and budgeted for at all levels and local councils should put in place innovative funding mechanisms to support them. Continued demand of monetary incentives by CHWs means that the spirit voluntarism might not be sustainable in the long run. This finding is in agreement with other studies that found a positive relation between financial incentives and good performance. A case control study among female volunteers in Bangladesh revealed a strong correlation between financial incentives and performance [29] while a qualitative study among CHWs on the tuberculosis control programme in the Northern Cape province in South Africa showed lack of monetary incentives as a major cause of attrition [35].

Although cash incentives might lower attrition rates, increase productivity and accountability of CHWs, such reward systems can present unforeseen negative consequences depending on how they are handled. Such payments can undermine community support and since money is never enough, CHWs might inevitably demand for more money and benefits. For smooth implementation of community health interventions, monetary incentives should be reasonable, sustainable, regular and comparable across all CHWs.

### Methodological consideration

Recall bias may have occurred when asking CHWs when they last performed activities on the iCCM programme. However, since CHWs were asked to recall activities done within short time intervals (1 to 4 weeks), such bias did not affect the quality of data greatly.

Some of the performance attributes such as visiting and referring sick newborns are not always routine activities (may not be done weekly) and this could have affected the overall performance scores. Since an observation of CHWs treating children was not done because of time constraints and given the nature of their work (patients not always present), the performance scores they received in the study may not necessarily correspond to their performance while treating children.

## Conclusions

Majority of CHW’s performance was suboptimal. Performance was affected by gender, community support, supervision and drug availability. Strategies to improve drug supply, community support and support supervision from the formal health system are necessary to improve the performance of CHWs.
